# Associations of normal fasting glucose levels and of insulin resistance with degenerative rotator cuff tear: Normoglycemia and rotator cuff tear

**DOI:** 10.1186/s12891-023-06899-5

**Published:** 2023-12-16

**Authors:** Hyung Bin Park, Ji-Yong Gwark, Jaehoon Jung

**Affiliations:** 1 Department of Orthopaedic Surgery, Gyeongsang National University School of Medicine, Gyeongsang National University Changwon Hospital, 11 Samjeongja-ro Seongsan- gu, Changwon, 51472 Republic of Korea; 2 Gyeongsang institute of medical sciences, Gyeongsang national university, Jinju, Republic of Korea; 3 Division of Endocrinology, Department of Internal Medicine, School of Medicine, Gyeongsang National University, Gyeongsang National University Changwon Hospital, Changwon, Republic of Korea

**Keywords:** Rotator cuff tendon tear, Risk factors, Normoglycemia, Fasting glucose level, TG/HDL ≥ 3.5, Insulin resistance

## Abstract

**Background:**

The upper normoglycemic range has been proposed as a risk factor for degenerative rotator cuff tendon tear (RCT), and insulin resistance has been suggested as a risk factor for tendinopathy. However, no research has established their association with degenerative RCT in the general population. This study aimed to determine whether fasting glucose levels and insulin resistance are risk factors for degenerative RCT in the normoglycemic population and identify the risk range for fasting glucose.

**Methods:**

This study included 418 normoglycemic participants from a rural cohort. Participants completed questionnaires, physical exams, blood tests, and MRI evaluations of both shoulders. Insulin resistance was assessed using a triglyceride/high-density-lipoprotein (TG/HDL) ≥ 3.5. Logistic regression analysis was used to determine the association between fasting glucose level, TG/HDL ≥ 3.5, and other factors and degenerative RCT. The study calculated the areas under the receiver operating characteristic curve (AUC) to determine the more appropriate predicting value between the scale and categorical values of fasting glucose levels, and compared the AUCs using the DeLong method.

**Results:**

In the multivariable analyses, both scale and categorical values of fasting glucose levels, and TG/HDL ≥ 3.5 were significantly associated with degenerative RCT. Fasting glucose levels ≥ 90.5 mg/dL (OR: 3.87, 95% CI: 2.10–7.06) in scale value and 90–99 mg/dL (OR: 4.13, 95% CI: 2.87–8.12) in categorical value were significantly associated with degenerative RCT (*P* < .001). The AUC of the scale value of fasting glucose levels ≥ 90.5 mg/dL was 0.68. The AUC of the categorical value of fasting glucose levels of 90–99 mg/dL was 0.70. Because of the significantly larger AUC of the categorical value of fasting glucose levels of 90–99 mg/dL, those fasting glucose levels were determined to be independently associated with degenerative RCT (P < .001).

**Conclusions:**

High fasting glucose levels within the normal range may link to increase insulin resistance and risk of degenerative RCT. Normoglycemic levels of 90–99 mg/dL and insulin resistance may be risk factors for degenerative RCT.

**Level of evidence:**

Level III, prognostic study.

**Supplementary Information:**

The online version contains supplementary material available at 10.1186/s12891-023-06899-5.

## Introduction

Rotator cuff tendon tear (RCT) is the most common shoulder disease entity that is associated with high healthcare costs in modern developed countries [1, 2]. Both intrinsic and extrinsic theories have been proposed to explain the causes of rotator cuff tendon degeneration [3–6]. While the RCT is believed to have multifactorial causes, its precise causes remain unidentified. Within this context, metabolic factors have been scrutinized as potential risk contributors to degenerative RCT [7–11]. Several metabolic factors, including dyslipidemia, metabolic syndrome, and diabetes, have been reported as risks associated with RCT [7, 9, 10, 12]. Notably, among these metabolic factors, diabetes has emerged as a consistent risk factor for degenerative RCT across various studies [13, 14]. Fasting glucose levels are an established diagnostic tool for diabetes or prediabetes. Categories of glycemia have been determined, by the American Diabetes Association, according to fasting glucose levels, thus: normoglycemia (< 100 mg/dL), prediabetes (100–125 mg/dL), and diabetes (≥ 126 mg/dL) [15]. In view of the incidence of diabetes-related complications and comorbidities in some groups among normoglycemic individuals, several studies have suggested that the currently accepted diagnostic criteria for hyperglycemia, which are hyperglycemic markers of fasting glucose, 2-hour glucose after an oral glucose load, and HbA1c, are late diagnostic criteria [16, 17]. There is growing concern that some chronic diseases might already be progressing, at a subclinical stage, long before a clinical diagnosis is made [16]. Even after controlling for other risk factors, recent studies suggest that, as fasting glucose levels increase, even while remaining within the currently accepted normoglycemic range (< 100 mg/dL), diabetes and cardiovascular disease risks also increase [18–20]. One study has reported that fasting glucose levels which are at the high end of the normal range are an associated factor for degenerative RCT [21]. However, that study enrolled as study subjects only hospitalized patients who had received rotator cuff repair or meniscectomy. Additionally, that study included patients whose fasting glucose levels were in the prediabetic range, with the case group ranging from 78 to 123 mg/dL and the control group ranging from 60 to 124 mg/dL. No study has, as yet, evaluated fasting glucose level as a risk factor for degenerative RCT in the general population or suggested a range of fasting glucose levels associated with that risk. One study reported that insulin resistance is a risk factor for Achilles tendinopathy [22]. However, there is little information as to whether insulin resistance is a risk factor for degenerative RCT. In view of the high healthcare costs for degenerative RCT management, a risk factor analysis pertaining to degenerative RCT prevention is valuable. This study hypothesized that increments of fasting glucose levels within the normoglycemic range and insulin resistance are associated with degenerative RCT. This study’s purposes were to determine, for the normoglycemic population, whether fasting glucose level and insulin resistance can be risk factors for degenerative RCT and, if so, the risk range for fasting glucose.

## Patients and methods

### Study Design and setting

This cross-sectional study’s data were derived from a cohort drawn from rural communities. This cohort consisted of 1,149 volunteers who had agreed to participate in a musculoskeletal disease investigation from June 2013 to December 2015. The data used in this study were derived from these subjects, who answered questionnaires and who underwent physical examinations, blood tests, simple radiographic evaluations of bilateral shoulders (true anterior-posterior, axillary lateral, and outlet views), and bilateral shoulder MRIs. This study was conducted based on the data collected from these examinations.

**Fig. 1:**
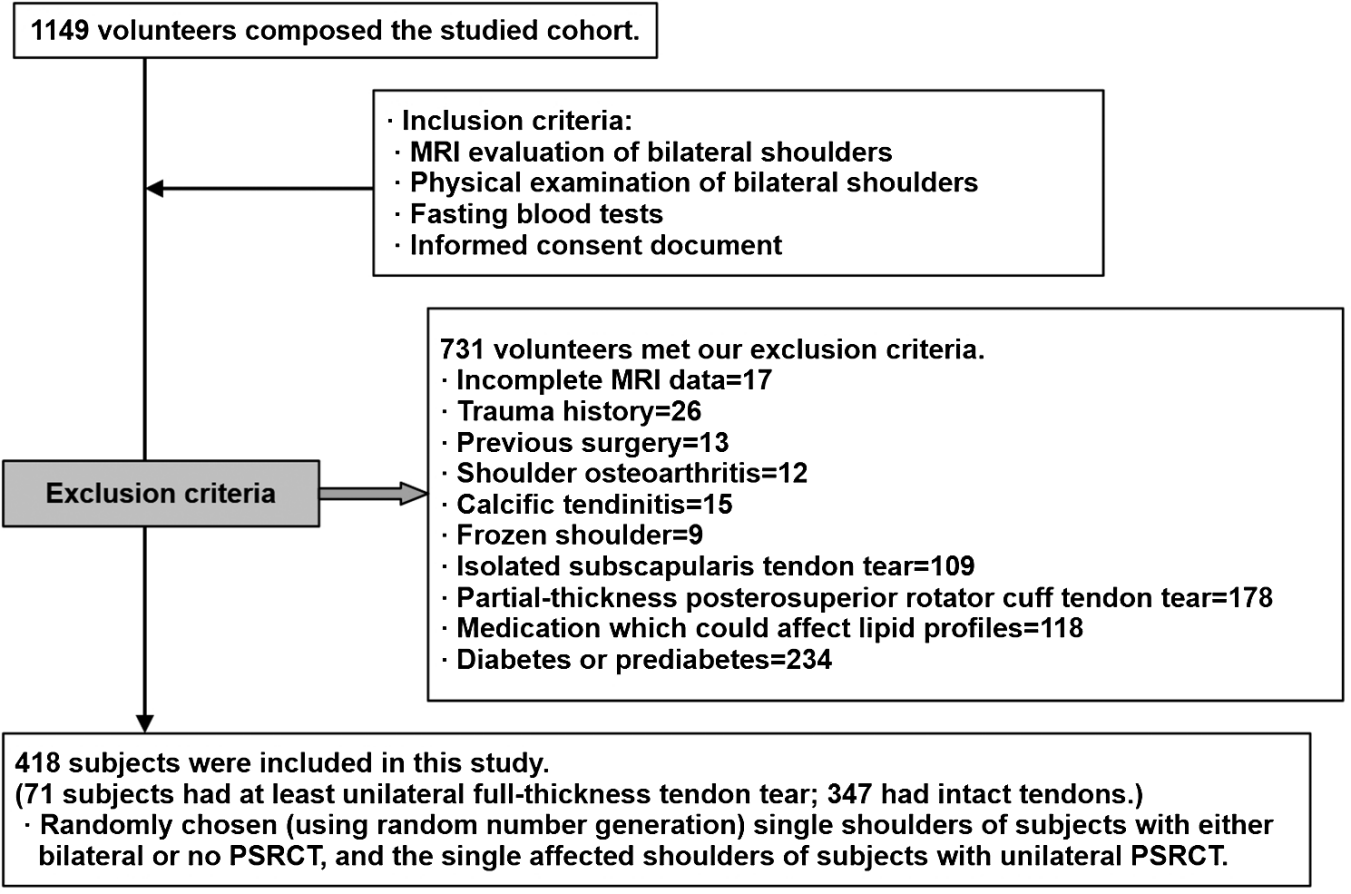
Flowchart for exclusion and inclusion criteria for this study. All 418 subjects met our inclusion and exclusion criteria

For the following reasons, 731 subjects were excluded from this study. 17 subjects did not participate in shoulder MRI studies. 362 subjects with at least one shoulder affected by trauma, previous surgery, osteoarthritis, isolated subscapularis tendon tear, or partial-thickness posterosuperior RCT were excluded because of this study’s primary focus on degenerative full-thickness posterosuperior RCT. 24 subjects with calcific tendinitis or frozen shoulder were excluded because both disease entities are known to be associated with abnormal glucose metabolism [23, 24]. 118 subjects had medications which could affect lipid profiles related to an insulin resistance marker (triglyceride/high-density lipoprotein). 210 subjects were excluded because they had diabetes or prediabetes, according to a laboratory test of fasting glucose level or a medical history (Fig. 1). Because this study’s methods involved the inclusion of only one shoulder per subject, one shoulder of each subject who had RCT in both shoulders or in neither shoulder was randomly included from the study. The selection for inclusion was made, using random number generation by the Excel program (version 2013, Microsoft, Redmond, WA, USA). If a subject had RCT in only one shoulder, only the involved shoulder was included. Therefore, this study enrolled 418 shoulders belonging to 418 subjects.

### Diagnosis of rotator cuff tear

This study only included full-thickness RCT. That was diagnosed primarily on the basis of individual MRI findings in which high signal intensity passed through the entire thickness of the tendon. When high signal intensity within the cuff tendon extended only to its bursal or articular surface, the tear was diagnosed as partial-thickness and excluded from this study [25]. For this study, by the use of a 1.5-T scanner (Siemens Medical Systems), these four sequences of MRI were obtained: oblique sagittal T1-weighted spin-echo, oblique sagittal T2-weighted turbo-spin-echo (TSE), oblique coronal T2-weighted TSE with fat saturation, and axial T2-weighted TSE with fat saturation. Each imaging sequence was obtained with a slice thickness of 3 mm, a field of view of 15.9 to 18.0 cm, and 1 excitation.

### Studied factors

The evaluated demographic factors were age and gender; the physical factors were height, weight, waist circumference, and dominant side; the social factors were smoking, alcohol consumption, and participation in labor according to the subjects’ occupations. The age factor was divided into five categories: < 40, 40 to 49, 50 to 59, 60 to 69, and ≥ 70 years. The investigated comorbidities were hypertension, metabolic syndrome (MetS), and hyper- and hypo-thyroidism. Hypertension was diagnosed according to previous medical history or the detection of blood pressure ≥ 140 mmHg for systolic or ≥ 90 mmHg for diastolic pressure [26]. Clinical identification of MetS was based on the presence of at least 3 of these 5 criteria: fasting glucose ≥ 100 mg/dL or use of anti-diabetic medication; systolic blood pressure ≥ 130 mmHg or diastolic blood pressure ≥ 85 mmHg, or taking antihypertensive medication; serum triglyceride (TG) ≥ 150 mg/dL; high-density lipoprotein (HDL) < 40 mg/dL for men or < 50 mg/dL for women; and waist circumference ≥ 90 cm for men or ≥ 85 cm for women [27]. The waist circumference criteria for obesity were adjusted according to Korean norms [28]. This study excluded subjects who had a fasting glucose level ≥ 100 mg/dL or who were users of medications for diabetes. Therefore, MetS was diagnosed in the presence of 3 of the 4 remaining MetS criteria, without the possibility of having subjects who had fasting glucose ≥ 100 mg/dL or who were using medications for diabetes. Prior diagnoses of hyper- and hypo-thyroidism were accepted. Additionally, new cases of hyper- and hypo-thyroidism were identified, according to thyroid function tests in which serum free thyroxine levels above 1.70 ng/dL indicated hyper-thyroidism and levels below 0.93 ng/dL indicated hypo-thyroidism [14].

Serum lipid profiles were evaluated as scale and categorical variables. Scale variables for serum lipids were total cholesterol, TG, low-density lipoprotein (LDL), HDL, and non-HDL. The categorical variable for serum lipids was the presence of any of the following dyslipidemias: hypercholesterolemia (total cholesterol ≥ 200 mg/dL), hyper-TGmia (TG ≥ 150 mg/dL), hyper-LDLemia (LDL ≥ 100 mg/dL), hypo-HDLemia (HDL < 40 mg/dL in male, < 50 mg/dL in female), and hyper-non-HDLemia (non-HDL ≥ 130 mg/dL) [29]. TG/HDL ≥ 3.5, the most frequently cited indicator for insulin resistance, was also evaluated [30].

### Statistical analyses

Odds ratios (ORs) with 95% confidence intervals (CIs) between RCT and various studied variables were determined, using logistic regression analyses. Univariate analyses were performed for all variables. Then, forward stepwise multivariable analyses were performed for all significantly associated variables with clinically significant effect sizes in the univariate analyses. Two different multivariable analyses were performed; one included scale value of fasting glucose levels, and the other included categorical value of fasting glucose levels. The multivariable logistic regression analyses were performed after assessment of multicollinearity, using variance inflation factors (VIF) and condition indexes. Multicollinearity was considered absent when both VIF and condition index were < 10 [31]. Each model with the lowest Akaike’s information criterion was chosen as the best of those resulting from the forward stepwise multivariable analyses [32]. Seven associated factors were included in each multivariable analysis to avoid overfitting [33]. The cut-off value for scale value of fasting glucose levels was determined, using the receiver operating characteristic (ROC) curve. Two separate forward stepwise multivariable analyses were performed for the cut-off value for scale value of fasting glucose levels and significant quartiles of categorical value of fasting glucose levels, using only the significant variables that had resulted from the univariate analyses. Goodness of fit for each multivariable model was determined by the Hosmer-Lemeshow, Stukel, and Osius-Rojek tests. The areas under the ROC curve (AUC) were calculated in order to choose, between the scale and the categorical values of fasting glucose levels, the more appropriate cut-off value. A comparison of the AUCs of the scale and the categorical values of fasting glucose levels was performed, using the DeLong method [34]. AUC values were classified as follows: values ≥ 0.90 were considered ‘excellent,‘ values from ≥ 0.80 to < 0.90 were categorized as ‘good,‘ values from ≥ 0.70 to < 0.80 were deemed ‘fair,‘ and values < 0.70 were classified as ‘poor [35].

All statistical analyses were performed using the SPSS software program (IBM SPSS Statistics for Windows, Version 24.0. Armonk, NY, USA: IBM Corp.), except the Stukel and the Osius-Rojek tests using R studio (rms software package in R, http://www.r-project.org). Significance of the logistic analyses was set at p < .05; of the Hosmer-Lemeshow test, at *P* > .05.

## Results

The study included 418 enrolled subjects with a mean age of 59 ± 9 years. Among these, 251 (59%) were female subjects with an average age of 59 ± 9 years, while 167 (40%) were male subjects with a similar mean age of 59 ± 9 years. The subjects were categorized into two groups: the ‘torn’ group, which consisted of 71 subjects, and the ‘intact’ group, comprising 347 subjects. Demographic data and the mean or median prevalence for each of the studied variables are summarized in Table 1.

**Table 1:** Summary of demographic data, prevalence, and mean or median for each studied variable

Studied variables	Enrolled subjects (n = 418)	Torn group (n = 71)	Intact group (n = 347)
Age (year)†	59 ± 9	62 ± 8	59 ± 9
< 40 years	5 (1%)	0 (0%)	5 (1%)
40 to 49 years	53 (13%)	3 (4%)	50 (14%)
50 to 59 years	165 (40%)	28 (39%)	137 (40%)
60 to 69 years	133 (32%)	24 (34%)	109 (31%)
≥ 70 years	62 (15%)	16 (23%)	46 (13%)
Male gender	167 (40%)	31 (44%)	136 (39%)
Waist circumference (cm)†	84 ± 9	88 ± 9	84 ± 9
< 85 in women, < 90 in men	247 (59)	32 (45%)	215 (62%)
85-89.9 (women), 90-94.9 (men)	79 (19%)	13 (18%)	66 (19%)
90-94.9 (women), 95-99.9 (men)	55 (13%)	14 (20%)	41 (12%)
≥ 95 (women), ≥ 100 (men)	37 (9%)	12 (17%)	25 (7%)
Dominant-side involvement	222 (53%)	46 (65%)	176 (51%)
Smoking	128 (31%)	22 (31%)	106 (31%)
Alcohol	273 (65%)	42 (59%)	231 (67%)
Manual labor	302 (72%)	61 (86%)	241 (70%)
Hypertension	132 (32%)	29 (41%)	103 (30%)
Metabolic syndrome	139 (33%)	38 (54%)	101 (29%)
Hyper-thyroidism	7 (2%)	3 (4%)	4 (1%)
Hypo-thyroidism	14 (3%)	1 (1%)	13 (4%)
Serum lipid levels (mg/dL)			
Cholesterol†	197 ± 35	198 ± 32	197 ± 35
TG*	108 (77 to 152)	121 (90 to 161)	106 (75 to 150)
LDL†	138 ± 33	140 ± 34	132 ± 31
HDL*	56 (47 to 67)	52 (46 to 65)	56 (47 to 68)
Non-HDL†	140 ± 35	145 ± 32	139 ± 36
Prevalence of dyslipidemia	339 (81%)	59 (83%)	280 (81%)
Hyper-cholesterolemia	195 (47%)	35 (49%)	160 (46%)
Hyper-TGmia	137 (33%)	28 (39%)	109 (31%)
Hyper-LDLemia	344 (82%)	67 (94%)	277 (80%)
Hypo-HDLemia	76 (18%)	25 (35%)	51 (15%)
Hyper-non-HDLemia	256 (61%)	57 (80%)	199 (57%)
TG/HDL	3 ± 2	3 ± 2	3 ± 2
TG/HDL ≥ 3.5	90 (22%)	26 (37%)	64 (18%)
Scale value of fasting glucose levels†	87 ± 6	92 ± 5	87 ± 7
Categorical value of fasting glucose levels			
< 85 mg/dL	116 (28%)	3 (4%)	113 (33%)
85–89 mg/dL	108 (26%)	12 (17%)	96 (28%)
90–94 mg/dL	105 (25%)	27 (38%)	78 (23%)
95–99 mg/dL	89 (21%)	29 (41%)	60 (17%)
Fasting glucose levels ≥ 90.5 mg/dL	173 (41%)	51 (72%)	122 (35%)

†Mean (standard deviation)

*Median (interquartile range)

TG, triglyceride; LDL, low-density lipoprotein; HDL, high-density lipoprotein

**Fig. 2:**
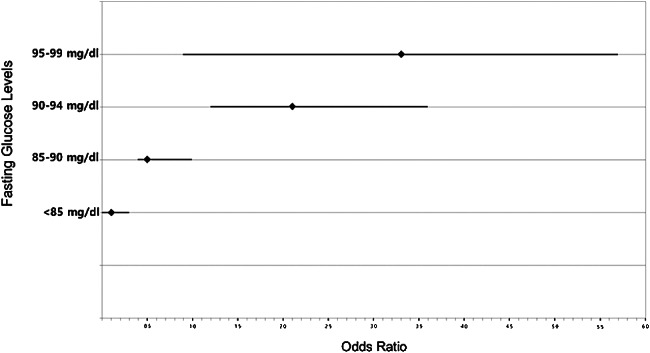
The forest plot shows the odd ratios for each quartile of fasting glucose levels and rotator cuff tear

This study’s univariate analyses identified the following variables as significantly associated with degenerative RCT: age, waist circumference, dominant side involvement, manual labor, MetS, LDL, hypo-HDLemia, hyper-non-HDLemia, TG/HDL ≥ 3.5, scale value of fasting glucose levels, and categorical value of fasting glucose levels (*P* < .032). Of the quartiles of fasting glucose categorical value, fasting glucose levels in the < 85 mg/dL quartile were significantly negatively associated with RCT (*P* < .001). In contrast, fasting glucose levels in the 90–94 mg/dL quartile or higher were significantly positively associated with RCT (*P* ≤ .007) (Fig. 2). The ORs and 95% CIs for all variables are summarized in Table 2.

**Table 2:** Strengths of associations between studied factors and degenerative rotator cuff tear, in univariate analyses

Studied variables	Odds ratio (95% CI)	*P* value
Age (year)	1.51 (1.14-2.00)	0.004
Waist circumference (cm)	1.49 (1.18–1.88)	0.001
Dominant-side involvement	1.79 (1.05–3.04)	0.032
Manual labor	2.68 (1.32–5.44)	0.006
Metabolic syndrome	2.81 (1.67–4.72)	< 0.001
LDLemia	4.23 (1.49-12.00)	0.007
Hypo-HDLemia	3.15 (1.78–5.58)	< 0.001
Hyper-non-HDLemia	3.03 (1.63–5.64)	< 0.001
TG/HDL ≥ 3.5	2.56 (1.47–4.45)	0.001
Scale value of fasting glucose levels (mg/dL)	1.21 (1.12–1.26)	< 0.001
Categorical value of fasting glucose levels†	2.72 (2.02–3.67)	< 0.001
< 85 mg/dL	0.09 (0.03–0.30)	< 0.001
85–89 mg/dL	0.53 (0.27–1.03)	0.062
90–94 mg/dL	2.12 (1.23–3.64)	0.007
95–99 mg/dL	3.30 (1.91–5.72)	< 0.001
Fasting glucose levels ≥ 90.5 mg/dL	4.70 (2.68–8.25)	< 0.001

†Fasting glucose levels were evaluated as a categorical value. Each level of glucose was assigned to one of five categories. The reference category was designated as < 80 mg/dL. CI, confidence interval; LDL, low-density lipoprotein; HDL, hypo-density lipoprotein; TG, triglyceride

Because there is multicollinearity between the scale value of fasting glucose levels and the categorical value of fasting glucose levels, multivariable analyses were performed in two separate ways (VIF: 11.20, condition index: 23.21).

The first multivariable analysis excluded the categorical value of fasting glucose levels, but included the other significant variables from the univariate analyses. According to that multivariable analysis, age, manual labor, MetS, hypo-HDLemia, hyper-Non-HDLemia, TG/HDL ≥ 3.5, and the scale value of fasting glucose levels were significantly associated with degenerative RCT. The ORs with 95% CIs for all variables, the VIFs, the condition indexes, the *P* values of the Hosmer-Lemeshow, Stukel, and Osius-Rojek tests are summarized in Table 3.

The second multivariable analysis excluded the scale value of fasting glucose levels, but included the other significant variables from the univariate analyses. According to that multivariable analysis, age, manual labor, MetS, hypo-HDLemia, hyper-Non-HDLemia, TG/HDL ≥ 3.5, and the categorical value of fasting glucose levels were significantly associated with degenerative RCT. The ORs with 95% CIs for all variables, the VIFs, the condition indexes, and the *P* values of the Hosmer-Lemeshow, of the Stukel, and of the Osius-Rojek tests are summarized in Table 3.

**Table 3:** Strengths of associations between studied factors and degenerative rotator cuff tear, in multivariable analyses

Including scale value of fasting glucose levels			Including categorical value of fasting glucose levels		
Studied variables	Odds ratio (95% CI)	*P* value	Studied variables	Odds ratio (95% CI)	*P* value
Age (year)	1.49 (1.08–2.07)	0.015	Age (year)	1.50 (1.09–2.08)	0.013
Manual labor	3.21 (1.39–7.40)	0.006	Manual labor	3.40 (1.47–7.80)	0.004
Metabolic syndrome	3.37 (1.83–6.22)	< 0.001	Metabolic syndrome	3.27 (1.80–5.97)	< 0.001
Hypo-HDLemia	2.18 (1.09–4.35)	0.027	Hypo-HDLemia	2.38 (1.20–4.72)	0.011
Hyper-Non-HDLemia	2.44 (1.22–4.89)	0.012	Hyper-Non-HDLemia	2.67 (1.34–5.29)	0.005
TG/HDL ≥ 3.5	2.20 (1.08–4.48)	0.030	TG/HDL ≥ 3.5	2.30 (1.16–4.59)	0.018
Scale value of fasting glucose levels	1.22 (1.11–1.26)	< 0.001	Categorical value of fasting glucose levels	2.03 (1.45–2.86)	< 0.001
			< 85 mg/dL	0.27 (0.11–0.58)	0.001
			85–89 mg/dL	0.83 (0.45–1.58)	0.618
			90–94 mg/dL	1.88 (1.04–3.28)	0.033
			95–99 mg/dL	3.08 (1.26–7.34)	0.006
VIF and condition index	2.80 and 5.17		VIF and condition index	2.67 and 5.02	
*P* value of Hosmer-Lemeshow, Stukel, and Osius-Rojek test	0.371, 0.298, and 0.412		*P* value of Hosmer-Lemeshow, Stukel, and Osius-Rojek test	0.416, 0.375, and 0.403	

CI, confidence interval; HDL, hypo-density lipoprotein; TG, triglyceride; VIF, variance inflation factor

**Fig. 3:**
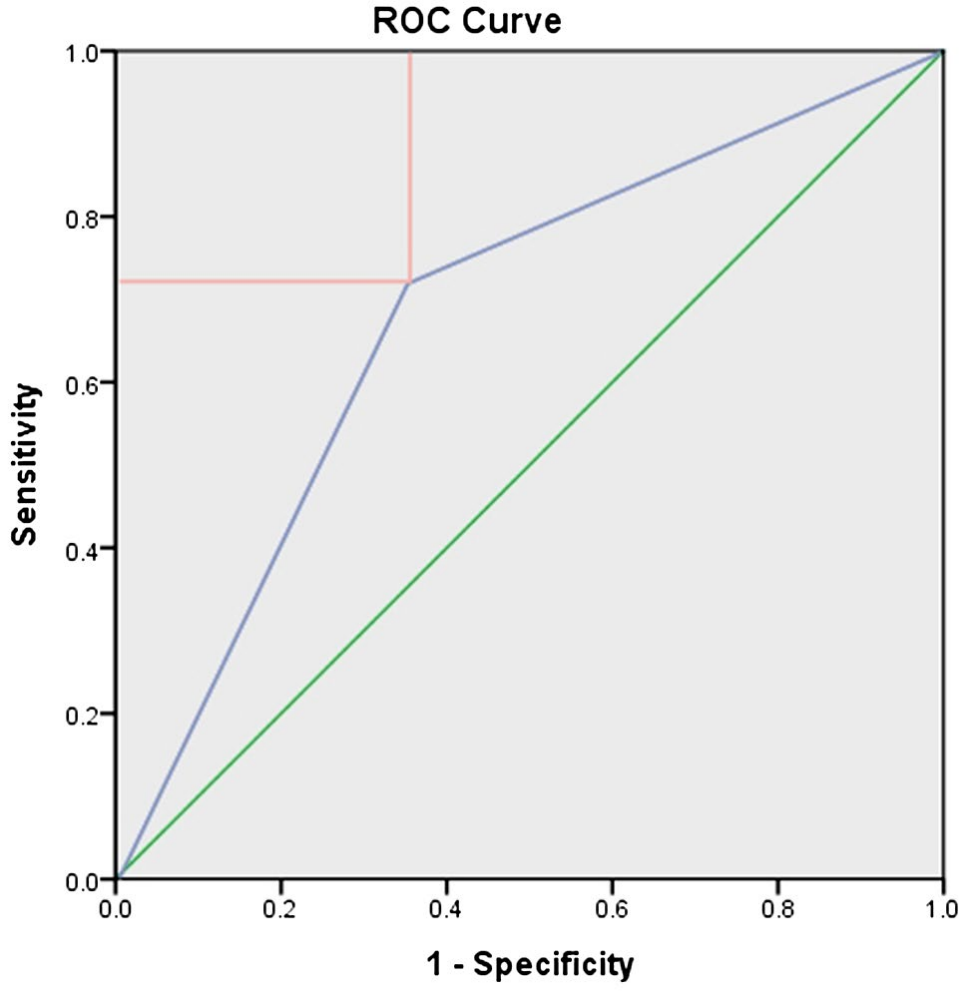
Receiver operating curve analysis for the cut-off value of fasting glucose level. Area under curve was 0.68 (95% CI 0.62 to 0.75; p < .001). Cut-off value was 90.5 mg/dL (sensitivity 72%, specificity 65%, positive predictive value 29%, and negative predictive value 92%)

The cut-off value of fasting glucose levels for significant association with degenerative RCT was found to be 90.5 mg/dL. Fasting glucose level ≥ 90.5 mg/dL had a “poor” performance rating for predicting degenerative RCT, with an AUC of 0.68 (95% CI [0.62–0.75]; *P* < .001) [36]. The sensitivity, specificity, positive predictive value, and negative predictive value were 72%, 65%, 29%, and 92%, respectively (Fig. 3). Fasting glucose level ≥ 90.5 mg/dL (OR, 3.87 [95% CI, 2.10–7.06]; *P* < .001) was significantly associated with RCT in multivariable analysis (Table 4). The ORs with 95% CIs for all variables, the VIFs, the condition indexes, and the *P* values of the Hosmer-Lemeshow, of the Stukel, and of the Osius-Rojek tests are summarized in Table 4.

**Table 4:** Strengths of associations between studied factors and rotator cuff tear, in multivariable analyses

Including fasting glucose levels ≥ 90.5 mg/dL			Including fasting glucose levels (90–99 mg/dL)		
Studied variables	Odds ratio (95% CI)	*P* value	Studied variables	Odds ratio (95% CI)	*P* value
Age (year)	1.53 (1.10–2.12)	0.012	Age (year)	1.44 (1.03–2.02)	0.030
Manual labor	3.55 (1.52–8.30)	0.002	Manual labor	3.71 (1.56–8.87)	0.003
Metabolic syndrome	3.70 (1.98–6.93)	< 0.001	Metabolic syndrome	3.22 (1.70–6.05)	< 0.001
Hypo-HDLemia	2.43 (1.22–4.84)	0.012	Hypo-HDLemia	2.59 (1.26–5.29)	0.009
Hyper-non-HDLemia	2.52 (1.25–5.05)	0.009	Hyper-Non-HDLemia	2.25 (1.11–4.58)	0.028
TG/HDL ≥ 3.5	2.21 (1.10–4.50)	0.025	TG/HDL ≥ 3.5	2.20 (1.11–4.36)	0.024
Fasting glucose levels ≥ 90.5 mg/dL	3.87 (2.10–7.06)	< 0.001	Fasting glucose levels (90–99 mg/dL)	4.13 (2.87–8.12)	< 0.001
VIF and condition index	1.36 and 6.71		VIF and condition index	1.42 and 3.61	
*P* value of Hosmer-Lemeshow, Stukel, and Osius-Rojek test	0.268, 0.387, and 0.409		*P* value of Hosmer-Lemeshow, Stukel, and Osius-Rojek test	0.412, 0.408, and 0.258	

CI, confidence interval; HDL, hypo-density lipoprotein; TG, triglyceride; VIF, variance inflation factor

**Fig. 4:**
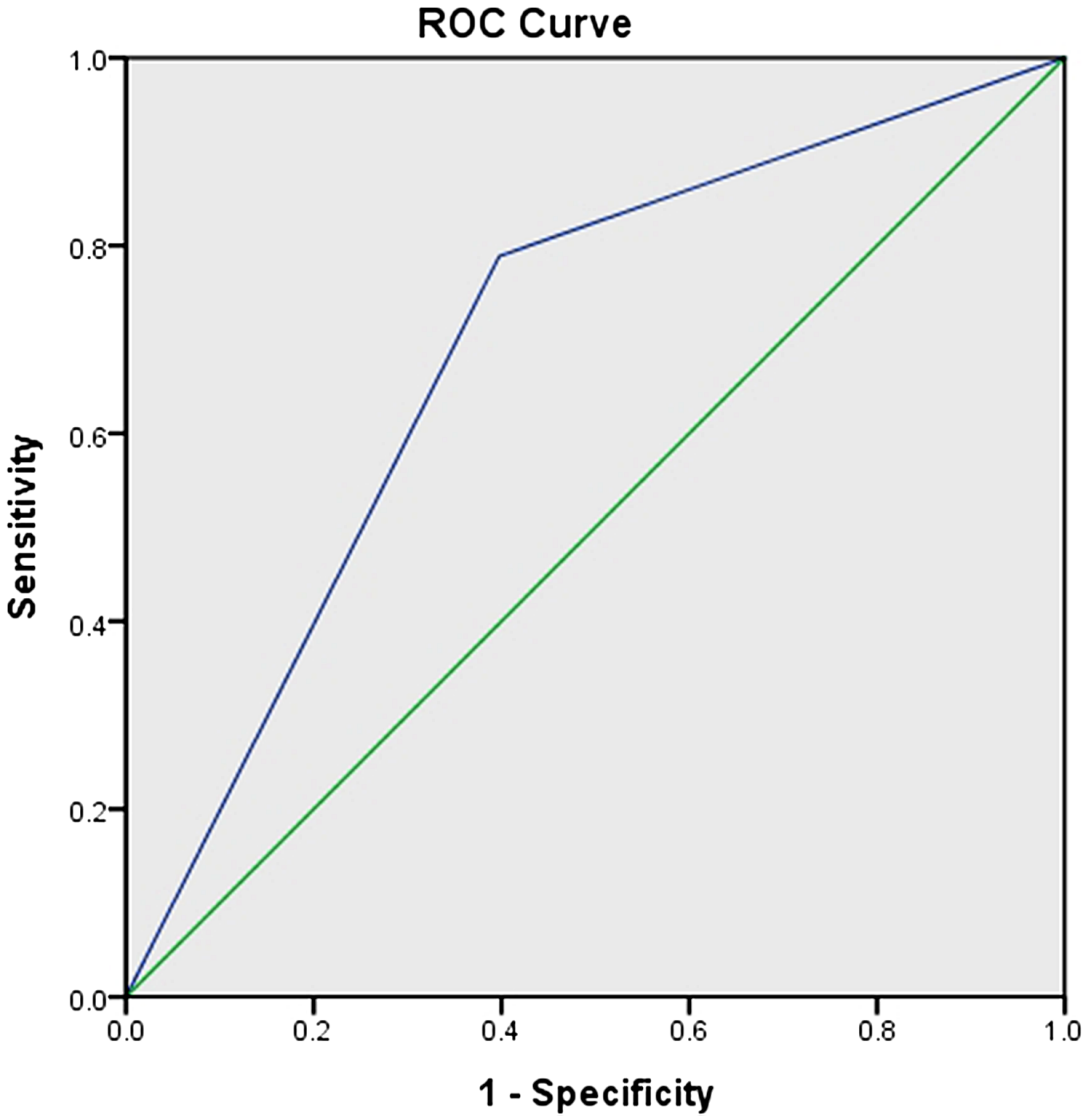
Receiver operating curve analysis for the categorical value of fasting glucose level (90–99 mg/dL). Area under curve was 0.70 (95% CI [0.63–0.76]; p < .001). The sensitivity, specificity, positive predictive value, and negative predictive value were 79%, 60%, 29%, and 93%, respectively

The categorical values of fasting glucose levels in the 90–94 mg/dL and 95–99 mg/dL quartiles were significantly positively associated with RCT (*P* < .001). The categorical value of fasting glucose levels of 90–99 mg/dL earned a “fair” performance rating for predicting RCT, with an AUC of 0.70 (95% CI [0.63–0.76]; *P* < .001) [36]. The sensitivity, specificity, positive predictive value, and negative predictive value were 79%, 60%, 29%, and 93%, respectively (Fig. 4). The ORs with 95% CIs for all variables, the VIFs, the condition indexes, and the *P* values of the Hosmer-Lemeshow, of the Stukel, and of the Osius-Rojek tests are summarized in Table 4.

The 0.70 AUC of the categorical value of fasting glucose levels of 90–99 mg/dL was significantly larger than the 0.68 AUC of fasting glucose levels ≥ 90.5 mg/dL; therefore, fasting glucose levels of 90–99 mg/dL were determined to be independently associated with degenerative RCT (P < .001).

## Discussion

This study supports the hypothesis that increments of fasting glucose levels within the normoglycemic range and insulin resistance are associated with degenerative RCT. This study found that both scale and categorical values of fasting glucose levels were significantly associated with degenerative RCT. The cut-off value of fasting glucose level was 90 mg/dL. TG/HDL ≥ 3.5 was also a significantly associated factor for degenerative RCT. This study confirmed that age and manual labor are risk factors for degenerative RCT, as has been frequently reported [37, 38].

This study found that fasting glucose levels, although in the normoglycemic range, are associated with degenerative RCT. A previous study reported that patients with degenerative RCT showed significantly higher fasting glucose levels within the normoglycemic range than the control group [21]. The current study supports the previous study’s finding that a fasting glucose level in the high-normal range is a risk factor for degenerative RCT. Hyperglycemia is known to be a risk factor for tendinopathy, RCT, and retear after rotator cuff repair [39]. However, the molecular mechanism of tendinopathy induced by hyperglycemia has not been completely determined. One proposed mechanism is that hyperglycemia induces oxidative stress and cytokine production, which lead to inflammation and tissue damage in various organs [40, 41]. Another proposed mechanism is that hyperglycemia induces an alteration of collagen structure through a glycation process [42–44]. Another suggested mechanism is that hyperglycemia produces a reduction in proteoglycan levels related to decreased synthesis or sulfation of glycosaminoglycans [45]. Most studies that have evaluated hyperglycemic effects on tendon degeneration have focused on diabetes. However, no study has investigated the level above which fasting glucose negatively affects rotator cuff tendon homeostasis.

This study indicated that fasting glucose levels of 90–99 mg/dL, although in the normoglycemic range, are associated with degenerative RCT. Several studies have reported that subjects are usually exposed to long-standing hyperglycemia or insulin resistance before being diagnosed with diabetes [46]. Several studies have reported that the risk of developing diabetes is higher for those in the 90–94 mg/dL quartile for fasting glucose levels than for those with levels < 85 mg/dL [18, 47]. One study has reported that frozen shoulder is positively associated with fasting glucose levels of 90–99 mg/dl, which are in the normoglycemic range [48]. Another study has suggested that high fasting glucose levels that are yet within the normal range have adverse physical effects and that the safe upper margin for fasting glucose is < 90 mg/dL [49]. Although the molecular mechanism involved in the effect of the upper normoglycemic range on rotator cuff tendinopathy has not yet been determined, these previous results and the current study’s findings suggest that maintaining fasting glucose levels below the proposed risk level could help reduce the risk of RCT and its related social and healthcare costs.

The TG/HDL ratio has been known as a simple way to identify apparently healthy individuals who are insulin resistant [30, 50]. Among several studies regarding the cut-off value for that ratio, which is affected by racial or ethnic background, age, and sex, TG/HDL ≥ 3.5 is the most frequently cited cut-off value [30, 50, 51]. In this study, TG/HDL ≥ 3.5 was significantly associated with degenerative RCT. One study, using TG/HDL as the parameter, reported that insulin resistance is a risk factor for Achilles tendinopathy [22]. Insulin resistance is a characteristic feature of hyperglycemia, which increases significantly with increasing fasting glucose level in the normoglycemic range [52, 53]. That previous study’s results and the current findings suggest that fasting glucose levels above a certain level within the currently accepted normoglycemic range may be linked to increased insulin resistance, which is associated with elevated risk of degenerative RCT.

This study indicated that MetS, hypo-HDLemia, and hyper-non-HDLemia are significantly associated factors for degenerative RCT. MetS, which is also known as insulin resistance syndrome, is a well-known risk factor for various degenerative diseases [54, 55] and has been reported to have an association with osteoarthritis [56, 57], Achilles enthesopathy [58], rotator cuff tendinopathy [59], and RCT [9]. Hypo-HDLemia, a constituent of MetS, has been reported as an associated factor for degenerative RCT [9]. Hyper-non-HDL involves inflammatory lipoproteins, which have been reported as involved in many chronic inflammatory cardiovascular diseases [60, 61]. In the musculoskeletal system, hyper-non-HDL has been reported as an associated factor for primary frozen shoulder [62, 63]. However, it is uncertain whether any study has determined that hyper-non-HDLemia is involved in degenerative RCT. Further study is needed to clarify the role of hyper-non-HDLemia in rotator cuff tendon degeneration or tear. Insulin resistance is known to play an essential role in the development of MetS, including in dyslipidemia, hypertension, and altered glucose metabolism [64]. These findings suggest that an association between degenerative RCT and fasting glucose levels in the high-normal range may be related to insulin resistance. The role of insulin resistance in rotator cuff tendon degeneration or tear is a subject for further study.

There are certain limitations associated with the present study that should be acknowledged. One of these limitations is the use of a single measurement of fasting glucose level, a common approach also employed by previous relevant studies [21, 47]. Ideally, multiple fasting glucose level measurements would have been taken to monitor daily fluctuations. However, individuals with diabetes or impaired fasting glucose, known to exhibit more fluctuations in fasting glucose levels than normoglycemic persons, were excluded from this study [65]. Consequently, any fluctuations occurring within the normoglycemic range would have affected both the control and study groups equally, as they were composed of individuals from the same ethnic group. As a result, the potential biases stemming from daily fluctuations and racial differences were minimized. Because this study focused on degenerative full-thickness RCT, subjects with partial-thickness posterosuperior RCT were excluded. To minimize the compound variable effect, subjects being medicated with any lipid-lowering drug were excluded. It is possible that those exclusions introduced a bias in the representation of the characteristics of the studied cohort. However, similar results were obtained from supplemental analyses that were conducted without those two exclusions (Supplemental tables). Therefore, the potential for bias because of those exclusions was small and, in view of the study’s purpose, appropriate. Although this is a cohort study, it could only include, without considering randomization, subjects who had volunteered; as primarily agricultural workers, their characteristics may not be generalizable to the entire population. As a cross-sectional study, this research cannot be used to evaluate the cumulative effects of various fasting glucose levels. Additionally, TG/HDL ≥ 3.5, not the oral glucose tolerance test or the hyperinsulinemic euglycemic clamp, was used to indicate insulin resistance.

## Conclusions

Normoglycemic fasting glucose levels of 90–99 mg/dL and insulin resistance are potential risk factors for degenerative RCT.

### Electronic supplementary material

Below is the link to the electronic supplementary material.

Supplementary Material 1

## Data Availability

The datasets used and/or analysed during the current study available from the corresponding author on reasonable request.
