# Supplementary material for: Associations of normal fasting glucose levels and of insulin resistance with degenerative rotator cuff tear: Normoglycemia and rotator cuff tear
Source: BMC Musculoskelet Disord. 2023 Dec 16;24:973. doi: 10.1186/s12891-023-06899-5 (PMC10724963; doi:10.1186/s12891-023-06899-5)
Supplement: Supplementary file 1 — Supplementary Material 1 [file 12891_2023_6899_MOESM1_ESM.doc]

| **Supplemental Table I** Strengths of associations between studied factors and degenerative rotator cuff tear, in univariate analyses (without excluding partial-thickness posterosuperior RCT and lipid-lowering drugs) | | |
| --- | --- | --- |
| Studied variables | Odds ratio (95% CI) | *P* value |
| Age (year) | 1.45 (1.12-1.86) | .003 |
| Waist circumference (cm) | 1.50 (1.17-1.88) | .001 |
| Dominant-side involvement | 1.77 (1.03-3.02) | .030 |
| Manual labor | 2.54 (1.26-5.19) | .003 |
| Metabolic syndrome | 2.80 (1.67-4.71) | < .001 |
| LDLemia | 4.31 (1.51-12.01) | .006 |
| Hypo-HDLemia | 3.03 (1.75-5.42) | < .001 |
| Hyper-non-HDLemia | 3.00 (1.60-5.60) | < .001 |
| TG/HDL ≥ 3.5 | 2.45 (1.37-4.35) | .001 |
| Scale value of fasting glucose levels (mg/dL) | 1.17 (1.10-1.25) | < .001 |
| Categorical value of fasting glucose levels† | 1.98 (1.47-2.68) | < .001 |
| < 85 mg/dL | 0.18 (0.06-0.47) | < .001 |
| 85-89 mg/dL | 0.89 (0.51-1.57) | .691 |
| 90-94 mg/dL | 1.85 (1.15-3.06) | .023 |
| 95-99 mg/dL | 3.12 (1.78-5.61) | .002 |
| Fasting glucose levels ≥ 90.5 mg/dL | 3.52 (2.07-5.95) | < .001 |
| †Fasting glucose levels were evaluated as a categorical value. Each level of glucose was assigned to one of five categories. The reference category was designated as < 80 mg/dL. CI, confidence interval; LDL, low-density lipoprotein; HDL, hypo-density lipoprotein; TG, triglyceride | | |

| **Supplement table II** Strengths of associations between studied factors and degenerative rotator cuff tear, in multivariable analyses | | | | | |
| --- | --- | --- | --- | --- | --- |
| Including scale value of fasting glucose levels | | | Including categorical value of fasting glucose levels | | |
| Studied variables | Odds ratio (95% CI) | *P* value | Studied variables | Odds ratio (95% CI) | *P* value |
| Age (year) | 1.46 (1.05-2.03) | .012 | Age (year) | 1.52 (1.10-2.06) | .011 |
| Manual labor | 3.20 (1.39-7.40) | .006 | Manual labor | 3.42 (1.49-7.79) | .003 |
| Metabolic syndrome | 3.35 (1.81-6.20) | < .001 | Metabolic syndrome | 3.25 (1.80-5.88) | < .001 |
| Hypo-HDLemia | 2.15 (1.06-4.31) | .025 | Hypo-HDLemia | 2.40 (1.22-4.74) | .011 |
| Hyper-Non-HDLemia | 2.43 (1.21-4.89) | .012 | Hyper-Non-HDLemia | 2.68 (1.34-5.27) | .004 |
| TG/HDL ≥ 3.5 | 2.22 (1.10-4.49) | .025 | TG/HDL ≥ 3.5 | 2.31 (1.17-4.57) | .017 |
| Scale value of fasting glucose levels | 1.21 (1.13-1.28) | < .001 | Categorical value of fasting glucose levels | 2.05 (1.46-2.88) | < .001 |
|  |  |  | < 85 mg/dL | 0.25 (0.10-0.55) | .001 |
|  |  |  | 85-89 mg/dL | 0.83 (0.45-1.55) | .608 |
|  |  |  | 90-94 mg/dL | 1.86 (1.04-3.28) | .035 |
|  |  |  | 95-99 mg/dL | 3.10 (1.30-7.35) | .005 |
| VIF and condition index | 2.87 and 5.01 | | VIF and condition index | 2.61 and 5.15 | |
| *P* value of Hosmer-Lemeshow, Stukel, and Osius-Rojek test | 0.371, .298, and .419 | | *P* value of Hosmer-Lemeshow,  Stukel, and Osius-Rojek test | 0.410, .373, and .410 | |
| CI, confidence interval; HDL, hypo-density lipoprotein; TG, triglyceride; VIF, variance inflation factor | | | | | |

| **Supplement table III** Strengths of associations between studied factors and degenerative rotator cuff tear, in multivariable analyses | | | | | |
| --- | --- | --- | --- | --- | --- |
| Including fasting glucose levels ≥ 90.5 mg/dL | | | Including fasting glucose levels (90-99 mg/dL) | | |
| Studied variables | Odds ratio (95% CI) | *P* value | Studied variables | Odds ratio (95% CI) | *P* value |
| Age (year) | 1.55 (1.12-2.10) | .008 | Age (year) | 1.55 (1.15-2.18) | .008 |
| Manual labor | 3.51 (1.52-8.25) | .002 | Manual labor | 3.41 (1.50-7.75) | .002 |
| Metabolic syndrome | 3.65 (1.95-6.91) | < .001 | Metabolic syndrome | 3.25 (1.80-5.90) | < .001 |
| Hypo-HDLemia | 2.42 (1.21-4.81) | .010 | Hypo-HDLemia | 2.80 (1.42-5.52) | .003 |
| Hyper-non-HDLemia | 2.50 (1.23-5.00) | .007 | Hyper-Non-HDLemia | 2.83 (1.45-5.50) | .001 |
| TG/HDL ≥ 3.5 | 2.24 (1.10-4.52) | .026 | TG/HDL ≥ 3.5 | 2.25 (1.15-4.40) | .021 |
| Fasting glucose levels ≥ 90.5mg/dL | 3.77 (2.01-6.84) | < .001 | Fasting glucose levels (90-99 mg/dL) | 3.92 (2.16-7.12) | < .001 |
| VIF and condition index | 1.38 and 6.75 | |  | 1.56 and 4.19 | |
| *P* value of Hosmer-Lemeshow,  Stukel, and Osius-Rojek test | .281, .389, and .411 | |  | .425, .461, and .390 | |
| CI, confidence interval; HDL, hypo-density lipoprotein; TG, triglyceride; VIF, variance inflation factor | | | | | |
